# Development of an integrative cessation program for co-smokers of cigarettes and cannabis: demand analysis, program description, and acceptability

**DOI:** 10.1186/1747-597X-8-33

**Published:** 2013-09-12

**Authors:** Julia Becker, Ines Hungerbuehler, Oliver Berg, Maciej Szamrovicz, Andreas Haubensack, Adrian Kormann, Michael P Schaub

**Affiliations:** 1 Swiss Research Institute for Public Health and Addiction ISGF, University of Zurich, Konradstrasse 32, P. O. Box, 8031, Zurich, Switzerland; 2 Department and Institute of Psychiatry, School of Medicine, University of São Paulo, Rua Dr. Ovidio Pires de Campos, 785, Cerqueira Cesar, Sao Paulo, CEP 05403-903, Brazil; 3 Arud Centres for Addiction Medicine, Konradstrasse 32, Zurich, 8005, Switzerland; 4 Integrierte Suchthilfe Winterthur ISW, Tösstalstrasse 19, Winterthur, 8402, Switzerland

**Keywords:** Tobacco, Cannabis, Cessation, Integrative smoking cessation program, Cognitive behavioural therapy, Motivational interviewing, Program development, Acceptability, Co-smoking

## Abstract

**Background:**

Tobacco and cannabis use are strongly interrelated, but current national and international cessation programs typically focus on one substance, and address the other substance either only marginally or not at all. This study aimed to identify the demand for, and describe the development and content of, the first integrative group cessation program for co-smokers of cigarettes and cannabis.

**Methods:**

First, a preliminary study using expert interviews, user focus groups with (ex-)smokers, and an online survey was conducted to investigate the demand for, and potential content of, an integrative smoking cessation program (ISCP) for tobacco and cannabis co-smokers. This study revealed that both experts and co-smokers considered an ISCP to be useful but expected only modest levels of readiness for participation.

Based on the findings of the preliminary study, an interdisciplinary expert team developed a course concept and a recruitment strategy. The developed group cessation program is based on current treatment techniques (such as motivational interviewing, cognitive behavioural therapy, and self-control training) and structured into six course sessions.

The program was evaluated regarding its acceptability among participants and course instructors.

**Results:**

Both the participants and course instructors evaluated the course positively. Participants and instructors especially appreciated the group discussions and the modules that were aimed at developing personal strategies that could be applied during simultaneous cessation of tobacco and cannabis, such as dealing with craving, withdrawal, and high-risk situations.

**Conclusions:**

There is a clear demand for a double cessation program for co-users of cigarettes and cannabis, and the first group cessation program tailored for these users has been developed and evaluated for acceptability. In the near future, the feasibility of the program will be evaluated.

**Trial registration:**

Current Controlled Trials ISRCTN15248397

## Background

Tobacco and cannabis are interrelated in a unique, multi-dimensional manner, with some connecting mechanisms that are distinct from the co-use of drugs in general [1]. Two of them are the shared route of administration (i.e., both substances are smoked) and co-administration (“mulling”, i.e., adding tobacco to cannabis joints, or blunts, i.e., rolling cannabis in cigar paper). Mulling is the most common way of using cannabis in Europe [2]. Epidemiological data show that tobacco smoking is more prevalent among those who consume cannabis compared to the total population. In a study in the United States, 74% of the marijuana users smoked cigarettes compared to 29% of the nonusers [3]. On the other hand, cannabis use is more common among tobacco smokers than among tobacco abstainers. In the National Survey on Drug Use and Health (NSDUH) in the United States, the 30 days prevalence of cannabis use was 36% among tobacco smokers compared to 11% among non-smokers [4]. In a general population survey on tobacco use in Switzerland, cannabis use during the 12 months before the survey was reported by 28% of the adolescents who smoked tobacco daily compared to 9% and 2% of the adolescents who were ex- and never-smokers, respectively [5].

Investigations examining the initiation of use, the transition to regular use, and the cessation of tobacco and cannabis use exemplify this interrelation. Tobacco use can act as a gateway to cannabis use [6], but the reverse, i.e., cannabis use acting as a gateway to tobacco use, has also been observed [7,8]. Additionally, the probability of a transition from occasional to regular tobacco smoking and nicotine dependence is higher in smokers who also use cannabis [7,9]. Similarly, (adolescent) cannabis users who also smoke tobacco seem to be at higher risk for regular cannabis use and cannabis dependence in young adulthood compared with cannabis-only users [10].

Regarding the cessation of tobacco use, longitudinal observational studies have demonstrated that tobacco smokers who also consumed cannabis made fewer attempts to quit using tobacco [11] and were less likely to successfully quit using tobacco compared with tobacco-only smokers [12]. Furthermore, cessation programs that exclusively address tobacco consumption appear to be less effective for individuals who also consume cannabis [13,14]. A balancing effect is one problem that co-smokers may be confronted with when wanting to stop using only one of the substances. It has been shown that the cessation of one substance often co-occurs with an increased use of the other substance [15-17]. These findings highlight the importance of accounting for concurrent tobacco and cannabis use when planning and evaluating interventions.

Despite this evidence, current cessation programs typically focus on one substance while only addressing the other substance either marginally or not at all. To our knowledge, no integrative smoking cessation program (ISCP) targeting co-smokers of cigarettes and cannabis in a group setting has been designed.

However, results of concurrent treatments of tobacco and alcohol dependence [18,19] and tobacco and illicit substance use (e.g., opiates; [20]) have been published. Additionally, some brief interventions targeting multiple substance use have shown promising results [21-23]. These findings demonstrate that it is feasible to combine a tobacco cessation intervention with an intervention that targets a second substance. Compared with single interventions, double interventions do not necessarily overstrain participants and reduce abstinence rates; instead, they generate putatively better outcomes with regard to one or both targeted behaviours [24,25].

The separate treatment histories surrounding tobacco and cannabis may be explained by the different legal statuses of the two substances that are often the subject of political discourse and election campaigns. In Switzerland for example, tobacco is categorised as a licit substance, while cannabis is an illicit drug. The divisions of the Swiss government that deal with these substances are both organisationally and financially separated from each other and, currently, so is the funding for prevention programs and research projects. Another explanation for the lack of combined treatment for tobacco and cannabis use may be the historical development of treatment and prevention systems in many industrialised countries. Treatment of cannabis dependence and co-occurring mental health problems is provided by the psychiatric systems of many countries. In contrast, tobacco cessation is possible without the involvement of psychiatrists and is part of the more general public health systems [24,25] that typically involve general health supply services. In Switzerland, health insurance coverage differs between the substances; while cannabis treatment in psychiatric services is covered by basic health insurance, smokers themselves are required to pay for nicotine replacement therapy and courses for tobacco cessation.

In recent reviews, researchers have stressed the need to develop and evaluate combined interventions for tobacco and cannabis users [1,26,27]. Agrawal and colleagues found evidence that dual abstinence may predict better cessation outcomes and therefore suggested developing out-patient treatment models [1].

The aim of the current study was to develop an ISCP. This process was accomplished in three steps, which will be explicated in this report. First, a preliminary study clarified whether there was a demand for an ISCP. Second, after having identified the demand, explicit information regarding co-smokers’ attitudes towards tobacco and cannabis and the association between both substances was collected for use when developing an ISCP. Moreover, co-smokers’ relevant experiences regarding quitting one or both substances simultaneously were collected. Third, based on the information gained during the second step, an ISCP was developed tailored to co-smokers of cigarettes and cannabis. This program incorporates the established therapeutic principles and strategies of former tobacco and cannabis cessation programs and takes into account reasonable concepts and ideas from the ongoing discussion about the mechanisms underlying the co-use of tobacco and cannabis and potential dependency problems.

## Methods

### Preliminary study and demand analysis

The perception of and the need for an ISCP were explored with semi-structured qualitative interviews with addiction experts, qualitative age-specific user focus groups, and a quantitative online survey designed for current and former co-smokers. Qualitative data were analysed according to the coding procedures of Grounded Theory [28]. Quantitative data were examined with descriptive statistics and logistic regression analyses, which were conducted to identify predictors of readiness to simultaneously quit cigarettes and cannabis. First, bivariate logistic regression analyses were used to identify potential predictors. These predictors were then entered into one model. Next, non-significant variables (*p* ≥ .05) were removed successively from the multivariate model. The resulting model was verified by separately adding the excluded variables to the model to account for suppressor effects. Only significant predictors (*p* < .05) were retained in the final model. In these analyses, only the respondents currently smoking cigarettes and using cannabis were included. All quantitative analyses were conducted using PASW Statistics Version 18 and 20 (SPSS Inc., Chicago, IL, USA).

#### Expert interviews

Twelve addiction experts participated in the semi-structured interviews about the relationship between tobacco and cannabis use and the demand for and possible design of an ISCP. These addiction experts worked in research or were practicing psychotherapy, medicine, prevention, or epidemiology. The majority were known local experts in tobacco and/or cannabis use. The experts were reimbursed with 180 Swiss Francs (corresponding to about 167 US dollars in February 2010 when the interviews were conducted). Most of the experts emphasised a substantial relationship between tobacco and cannabis use that can cause problems, especially in the context of cessation attempts. For example, some experts observed that individuals who consumed both tobacco and cannabis increased their use of one substance when attempting to quit the other, which could lead to elevated risk of relapse. Quitting both substances simultaneously might prevent this balancing effect. Thus, the experts perceived a clear demand for an ISCP. Despite this demand, the experts assumed that few co-smokers would be ready to stop their tobacco and cannabis use simultaneously because smokers often perceived quitting tobacco use as a “loss” and probably would not be ready to additionally “give up” cannabis use.

Regarding the design of an ISCP, the experts favoured a group setting and suggested incorporating methods from cannabis treatment manuals into an established tobacco cessation program. The experts believed that integrating an additional substance into a tobacco cessation program would enhance its complexity (e.g., they expected a relatively high rate of participants with psychiatric comorbidities such as depression). Thus, an ISCP should offer comprehensive medical, psychiatric, and psychotherapeutic support for participants, on-demand additional single treatment sessions, and specific training for the course instructors that would aid in addressing the complexities and potential problems of double cessation.

The experts differed in their opinions about the appropriate age range of the participants. However, some experts suggested that co-smokers aged 25 years and above should be targeted because the experts expected a higher level of readiness to participate in an ISCP among this age group compared with younger co-smokers. This reasoning was based on the common assumption that cannabis use during adolescence is transient and thus less problematic and on the fact that family planning usually becomes more relevant at the age of 25 years. Two experts suggested separating groups by gender.

#### Focus groups with former and current co-smokers

The focus group discussions were conducted to gain in-depth information concerning users’ problems, experiences, and methods of coping with the issues that occurred during cessation attempts. Recruitment was organised via counselling facilities, and participants received financial reimbursement for participation. To be included in the focus group discussions, candidate participants had to self-report 1) past or current tobacco dependence, 2) past or current use of cannabis at least several times per week, and 3) at least one attempt to quit cigarette smoking, cannabis use, or both with formal treatment. As an incentive, focus group participants received 100 Swiss Francs in cash (corresponding to about 95 US dollars in April 2010 when the focus groups were conducted).

Similar to the experts, the 14 participants of the focus group discussions (10 adolescents aged 16 to 22 years and four adults aged 27 to 39 years) perceived a strong relationship between tobacco and cannabis use. Many of the participants reported experiences with the aforementioned balancing effect. However, the adolescents in particular demonstrated low willingness to quit cannabis and discussed their negative outcome expectancies concerning tobacco and cannabis cessation attempts (e.g., weight gain, sleeplessness, and increased alcohol consumption). The participants assumed that the general willingness of co-smokers to quit both substances would be low. However, due to the relationship between tobacco and cannabis use, they considered an ISCP to be useful.

Regarding the ISCP design, participants emphasised the importance of appropriate knowledge transfer concerning the interrelationship of the substances. The participants deemed the differences between potential participants with regard to their motivations to quit, aims, consumption patterns, and life situations to be relevant. Therefore, they suggested that all course participants should form a common goal. Furthermore, focus group participants indicated that there was a strong need for the development of appropriate relaxation and stress reduction methods.

#### Online survey with former and current co-smokers

Taking into account the information provided by the expert interviews and user focus groups, the online survey included questions concerning smoking behaviour, quitting experiences, and attitudes towards tobacco, cannabis, and an ISCP. Moreover, the online survey investigated the demand for an ISCP, co-smokers’ willingness to participate in such a program, and their readiness to quit both substances simultaneously. Former co-smokers were asked to indicate whether they would have been willing to participate in an ISCP and whether they would have been ready to quit tobacco and cannabis simultaneously. Recruitment was achieved through advertisements in internet forums on smoking, cannabis use, and health and via two social media platforms. Lotteries for a city trip, a tablet computer, and book vouchers were used to encourage participation.

The online survey began with 247 respondents who met the inclusion criteria of smoking both tobacco and cannabis either regularly at the time of the survey (current co-users, *n* = 109) or in the past (*n* = 138). Current co-use was defined as daily tobacco use and the use of cannabis during the past seven days before the survey. The survey was completed by 79.4% (196/247). Data from drop-outs were excluded in an item-wise manner. There were no significant differences between the drop-outs and the completers regarding age (*U* = 4908.50, *p* = .844), sex (χ^*2*^(1) = 0.368, *p* = .636), educational level (*U* = 4751.5, *p* = .581), or smoking frequency (tobacco: *U* = 4560.0, *p* = .289; cannabis: *U* = 4616.5, *p* = .397).

The respondents were between the ages of 14 and 88 years (*M* = 28.71, *SD* = 8.46), and 44.9% (111/247) were female. More than half of the respondents had previously attempted to stop smoking tobacco (74.7%, 183/245) and/or cannabis (51.2%, 124/242) at least once. Of those who had attempted to quit tobacco, 19 respondents (33.3%) increased their cannabis use after their tobacco cessation. More than half (51.6%) of those who had attempted to quit cannabis reported an increase in tobacco use.

As shown in Table 1, smokers’ potential and ex-smokers’ actual reasons for quitting differed significantly according to the substance (tobacco or cannabis). Reasons for quitting tobacco use were related to physical health aspects, whereas the most common reasons for cannabis cessation were problems with memory, concentration, motivation, and achievement. Respondents could also specify further reasons, which were not included in the list. The listed reasons were quite heterogeneous. However, several respondents mentioned the lack of desire to smoke tobacco and/or cannabis as a potential or actual reason to quit. For tobacco use, several respondents listed olfactory or gustatory reasons for quitting, and for cannabis use, some respondents mentioned that they experienced no effects or negative effects after the use of the substance as a reason for quitting.

**Table 1:** **Reasons for quitting tobacco and cannabis use among online survey respondents (*n*** **= 219)**

**Which have been or could be reasons for you to quit tobacco/cannabis? *Multiple answers possible.***	***n *(%) of respondents who checked each reason for quitting tobacco/cannabis**				**McNemar test**	
	**Tobacco**		**Cannabis**		**χ**^**2**^	***p***
Problems with health	145	(66.2)	102	(46.6)	22.33	< 0.001
Decreasing physical fitness	134	(61.2)	77	(35.2)	40.73	< 0.001
Pregnancy/starting a family	87	(39.7)	73	(33.3)	4.97	0.026
Financial reasons	70	(32.0)	57	(26.0)	2.82	0.093
Non-smoking partner	69	(31.5)	43	(19.6)	14.20	< 0.001
Feeling of being dependent/not free	113	(51.6)	79	(36.1)	13.28	< 0.001
Problems with memory or concentration	37	(16.9)	112	(51.1)	62.94	< 0.001
Problems with motivation or achievement	47	(21.5)	113	(51.6)	52.81	< 0.001
Mental health problems	39	(17.8)	100	(45.7)	53.73	< 0.001
Other	21	(9.6)	32	(14.6)	3.45	0.063

McNemar’s χ^2^ with continuity correction.

Table 2 shows attitudes towards the potential negative effects of tobacco and cannabis smoking. Compared to the analogous statements for cannabis smoking, a significantly higher proportion of respondents confirmed the statements concerning the negative effects of tobacco smoking.

**Table 2:** **Attitudes towards tobacco and cannabis use among online survey respondents of the preliminary study (*n*** **= 216)**

**Smoking tobacco/cannabis…**	**Tobacco**	**Cannabis**	**Wilcoxon signed-rank test**	
	***n *(%)**	***n *(%)**	***z***	***p***
…is harmful to my health	197 (91.2)	114 (52.8)	−8.38	< 0.001
…can cause lung cancer, heart diseases and other serious diseases	190 (88.0)	122 (56.5)	−7.52	< 0.001
…is addictive	185 (85.6)	81 (37.5)	−9.13	< 0.001
…promotes premature skin aging and harms one’s appearance	147 (68.1)	75 (34.7)	−8.43	< 0.001

Items could be answered with “I fully agree”, “I somewhat agree”, “I somewhat disagree”, or “I fully disagree”, *n* (%) for “I fully agree” responses are displayed.

Half of the respondents (124/247) thought that smoking tobacco and cannabis were interrelated (33.6% responded “yes probably”, and 16.6% responded “yes”). Furthermore, 67.6% (140/207) of the respondents affirmed the need for an ISCP (22.2% responded “yes”, and 45.5% responded “yes probably”).

However, of those who were currently smoking tobacco and using cannabis only 27.6% (29/105, 95% confidence interval [*CI*] = 0.20–0.37) stated that they felt ready to quit both substances simultaneously (15.6% responded “yes”, and 11.0% responded “yes probably”), and 41.4% (36/87, 95% *CI* = .31–.52) felt ready to participate in an ISCP (10.1% responded “yes”, and 22.9% responded “yes probably”).

Three predictors significantly predicted readiness to quit tobacco and cannabis simultaneously in a logistic regression analysis. Age was positively associated with readiness for simultaneous cessation (odds ratio [*OR*] = 1.11, 95% *CI* = 1.03–1.19, *p* = .005). Furthermore, using cannabis at least once a week predicted a lower likelihood of feeling ready to quit compared with using cannabis less frequently (*OR* = 0.12, 95% *CI* = 0.04–0.40, *p* = .001). Finally, partial or full agreement with the statement “Cannabis is harmful to my health” increased the likelihood of readiness to quit the substances simultaneously (*OR* = 4.13, 95% *CI* = 1.43–11.94, *p* = .009).

With regard to the program design, more than 70% (127/179) of the respondents considered an ISCP useful for individuals between 20 and 25 years and more than half of the respondents found it useful for those between 15 and 20 years (101/179) and for those between 25 and 30 years (98/179; multiple answers possible). In general, 69.9% (137/196) of the respondents preferred age-separated but only 14.3% (28/196) preferred sex-separated groups. The majority of the respondents (66.9%, 131/196) also suggested making the groups accessible only to smokers of both substances.

### Conceptualisation of the intervention

Given that both experts and co-smokers considered a therapeutic program for co-smokers as important and sensible, the development process was continued.

The intervention development proceeded as follows: first, literature was reviewed concerning effective interventions for tobacco and those for cannabis. Based on these findings and the results of the preliminary study, an interdisciplinary expert team composed of three psychologists, one social education worker, and three psychiatrists developed the group intervention. The team members had experience (and expertise) in research, therapeutic practice (single and group interventions for tobacco, alcohol, and illegal drugs), and/or the development of intervention programs for the treatment of substance abuse. The program development was an incremental and iterative process that provided the team members with multiple opportunities for feedback. The experts met eight times during the seven months before the beginning of the course. During and after the first implementation phase, the experts held two further meetings to adapt the manual for the second implementation phase.

Additionally, a thorough recruitment strategy was designed to reach as many co-smokers as possible and motivate them to participate in the ISCP. The recruitment process was conceptualised as an integral part of the ISCP and acted as an intervention in and of itself. Therefore, this recruitment strategy will be described in detail in the following chapter about the ISCP.

After the first implementation phase, the intervention was slightly refined based on the feedback of the course facilitators, some of whom were also part of the expert team. Given that there were only minor changes (i.e., the addition of a sixth course session that did not add content, as it only redistributed the course contents over six instead of five sessions), in the following chapter not both versions are described but only the adapted, second version.

### Description of the integrative cessation program for co-smokers of cigarettes and cannabis

#### Recruitment strategy

The recruitment strategy was planned extensively given that both the experts and focus group participants anticipated in the preliminary study that co-smokers would only show modest willingness to quit their tobacco and cannabis use simultaneously and to participate in an ICSP. To reach as many co-smokers as possible, information about the new course was spread via different channels.

First, a website (http://www.i-cut.ch) consisting of two parts was designed. The first part provided information about the course (i.e., content, structure, and dates) and the possibility to register for an information evening. The second part aimed to enhance co-smokers’ motivation to quit simultaneously and participate in the ISCP, primarily by providing information, offering a self-assessment with normative feedback, and using techniques adapted from motivational interviewing [29].

During the next step, a press release was issued. This step occurred only once at the beginning of the study and attracted a great deal of interest, which resulted in several reports in local newspapers and on radio and TV stations. Counselling centres for addiction prevention and treatment, psychiatrists, and health (care) centres in the canton of Zurich and bordering cantons helped spread flyers and leaflets that referred to the website for more information. Additionally, two social media platforms and an advertisement in the online edition of a popular free newspaper were used for online recruitment, also referring to the program website for more information.

The final step involved planning an information evening. Interested co-smokers could attend it without any obligation to participate in the course. The information evening provided the opportunity to ask questions that were answered by the course instructors, who introduced themselves and the course program and presented some background information. As central issues, they emphasised the association between tobacco and cannabis use and the potential physical harm of cannabis use, which was underestimated by co-smokers in the preliminary study. Additionally, instructors mentioned that co-smokers could participate together with co-smoking friends and partners to start the behavioural change together and support one another.

#### Course setting

Consistent with the findings from the preliminary study, the expert team considered an outpatient group-setting with 8 to 12 co-users of tobacco (who smoked at least one cigarette per day) and cannabis (who smoked at least once a week) per group as appropriate. The group-setting was preferred due to several general advantages of group therapy, such as cost-effectiveness (fewer treatment personnel are needed) and interpersonal processes (e.g., peer support and peer pressure) [30]. Additionally, in this new field of dual cessation of tobacco and cannabis use, the opportunity of group participants to share cessation experiences and strategies was considered especially important. Given that the co-smokers in the preliminary study preferred age-specific groups, we set the minimum age for participation in the ISCP at 20 years. Adolescents were excluded from this first version of the intervention for two reasons. First, being younger was associated with a decreased readiness to quit tobacco and cannabis simultaneously among the online survey respondents of the preliminary study. This finding is in line with the assumption of some of the interviewed experts in the preliminary study who expected greater readiness to participate among co-smokers aged 25 years and above. Second, an effective ISCP for adolescents should presumably differ from an ISCP designed for adults; for example, an ISCP for adolescents should account for school and family problems. Thus, the expert team decided to develop a basic program version for adults that could be adapted for adolescents if the basic version proved to be feasible. Separating groups by gender was considered but deemed to be unfeasible due to the expected low number of co-smokers who were ready for participation. Furthermore, the online survey respondents in the preliminary study clearly preferred age-specificity to gender-specificity.

Two local addiction treatment centres in the Swiss cities of Zurich and Winterthur offered the courses. Each course was guided by two course instructors, at least one of whom had to be a psychiatrist to guarantee the offer of prescription pharmacotherapy to reduce acute withdrawal symptoms or eventual exacerbations of severe psychiatric symptoms. The second instructor had to have experience in treating tobacco or cannabis smokers and could be from a different profession. The members of the expert team either guided the course sessions themselves or trained additional instructors to do so. All instructors received a therapist manual containing instructions for guiding the sessions and the information that was provided to the participants in their workbook (see below).

#### Course structure, content, and goals

**Figure 1:**
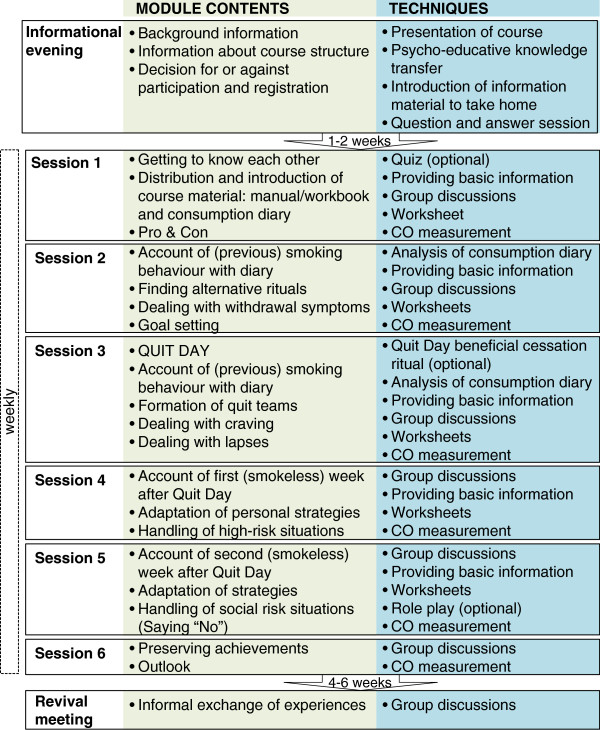
**Course structure, content, and techniques of the integrative cessation program for tobacco and cannabis co-smokers.** Legend: CO = carbon monoxide.

The experts interviewed during the preliminary study recommended using an established tobacco cessation program as the basis for the ISCP and combining it with cannabis-specific elements. We therefore utilised parts of the group tobacco cessation course used by the UK’s leading charity for gay men’s health (GMFA), which was evaluated by Harding and colleagues [31] and with which we were familiar, given that we culturally adapted and scientifically evaluated this program for Switzerland. We integrated elements from cannabis interventions that were ongoing under the supervision of members of the expert team. The resulting course was structured into six weekly sessions and one revival meeting that occurred approximately six weeks after the last session (Figure 1). Given anticipated recruitment problems, the expert team chose a small number of sessions to generate a low-threshold intervention. Each of the sessions lasted between 90 and 120 minutes. Additionally, course instructors offered each participant one individual counselling session on request.

Subsidiary elements of the course sessions were a smoking diary and workbook which were introduced and distributed to the participants during the first session. The workbook contained background information, an overview of the course structure and content, and work sheets to reflect on personal reasons for cessation and develop and write down personal strategies. The smoking diary was a small notebook that could easily be carried to constantly monitor consumption and thoughts, feelings, and actions associated with the use of one or both of the substances. The notebook should promote vigilance and self-examination. Furthermore, participants measured their expired carbon monoxide at every session to receive an immediate objective feedback on their therapy progress and for later program evaluation. They could enter their individual values in their notebooks and thereby monitor the changes in the values.

The main goal of the intervention was dual abstinence of tobacco and cannabis. The instructors promoted moderation of tobacco and/or cannabis use, for example, reducing smoking frequency or changing to a less harmful method of administration (e.g., consuming cannabis orally based on medical cannabis use recommendations for severe treatment resistant diseases) only when participants failed to quit several times during the course. This was done to avoid course dropout. Regarding the cessation sequence of tobacco and cannabis, the expert team supported a simultaneous cessation with one fixed quit date (Quit Day), when the participants were required to stop their tobacco and cannabis use, which is in line with Agrawal’s suggestions [1]. Before that date, participants could either maintain their normal use of tobacco and cannabis or start to reduce or stop one or both substances. Apart from the importance of having a common goal, i.e., stopping the use of both substances on a fixed quit date (at the latest) to foster group dynamics, this procedure was chosen for several reasons. First, the strong association between tobacco and cannabis use, in that each substance may act as a behavioural cue for the other [32], suggests that using neither substance is likely the easiest manner of quitting. In addition, this strategy has the advantage of experiencing only one withdrawal phase. Withdrawal symptoms may be stronger for the cessation of both substances compared with the symptoms for each substance alone, yet evidence suggests that this difference occurs for only a short duration and varies substantially between individuals [33]. For some quitters, withdrawal during dual abstinence may even be less severe than withdrawal from each substance individually [33].

#### Therapeutic principles

The course was primarily based on principles of motivational interviewing [29], self-control practices [34], the relapse-prevention model [35], and methods used in cognitive behavioural therapy that have been shown to be effective in the cessation of tobacco [36] and cannabis [37-39].

Although most of these principles and techniques were applied throughout the whole duration of the course, the emphasis on the application of each principle differed according to the Transtheoretical Model of Behavior Change [40]. Consequently, motivational enhancement strategies predominated in the early sessions of the intervention to address participants’ ambivalence about quitting and strengthen their motivation to change. In the following sessions, self-control practices were highlighted, such as an analysis of one’s own smoking behaviour with the help of the smoking diary. After the Quit Day, relapse prevention was the focus; relapse prevention focused on the development of personal strategies to avoid or cope with tobacco and cannabis use triggers.

Additionally, participants were encouraged to use medications that are typically used as first-line medications to increase long-term tobacco abstinence [36]. As some negative outcomes have been observed in bupropion studies with cannabis users [41,42], course instructors recommended varenicline and nicotine replacement therapy. Instructors provided information about these medications during the information evening, handed out leaflets that participants could take home, and indicated the corresponding information in the workbook that every participant received at the start of the course.

The intervention sessions that followed the information evening occurred in a group course setting. A meaningful part of every session was the group discussion. In these discussions, the participants could share experiences and problems, and support was provided both among the participants and from the instructors to the participants (i.e., intra-treatment support). Confidentiality was ensured, and instructors placed great value on providing an open, non-judgemental atmosphere. Additionally, the instructors promoted the formation of small quit teams, i.e., subgroups of two or three participants who supported each other, especially between the course sessions, such as during episodes of strong craving. Engagement in a Quit Team was optional and the formation and organization of the Quit Teams was not guided by the course instructors.

### Acceptability study of the integrative cessation program

We investigated the acceptability of the intervention among the participants and course instructors. Further analyses of smoking-related outcomes and utilisation will be conducted with the follow-up data in the near future.

#### Sampling and recruitment

Course participants were recruited with the above-mentioned methods. To be included in the intervention, participants had to use cannabis at least weekly, smoke tobacco (in addition to any tobacco used in joints) daily, be at least 18 years old, and be German literate. Participants were not reimbursed, but participation was free, which may have been attractive because, in Switzerland, tobacco cessation programmes usually require payment from the participants themselves.

Recruitment of course instructors began within the expert team that developed the intervention. As mentioned above, some experts also acted as course instructors. The experts also trained co-workers from their institutions to guide the courses.

#### Measurements and analyses

At the end of treatment, participants completed questionnaires that they received either at the end of the last session or by mail if they missed the last session. The questionnaire contained a set of items that measured the participants’ opinions toward the intervention in general and toward several components of the intervention. The instruction for the general items began as follows: “How would you evaluate the course regarding”. The instruction for the course component items began as follows: “How helpful were the following components for you?” All items were rated on a scale from 0 to 5, and higher values indicated more positive evaluations.

The course instructors received an analogous evaluation form after they had conducted the last course and indicated to what extent the course components were helpful to the participants.

In addition to descriptive statistics, the ratings for the general items were compared between participants of the first (five course sessions) and second implementation phases (six course sessions) using Mann–Whitney U tests. This comparison was not applied to the specific course components because their contents did not differ between the two phases.

#### Ethical approval

This intervention study was performed in compliance with the Declaration of Helsinki and was reviewed by the Ethics Committee of the Canton of Zurich, which did not declare any objections (KEK-StV-Nr.23/11). Participants signed an informed consent form prior to the first group therapy session.

## Results

### Sample characteristics

Over nine months, a total of 77 co-smokers participated in seven groups with six to 13 participants each. Of these participants, 59 (76.6%) answered at least one item of the course evaluation. Of these 59, 31 participated during the first and 28 during the second implementation phase. The majority of the respondents (71.2%) were male, their mean age was *M* = 34.0 (*SD =* 8.1) years, and most (84.7%) were Swiss. Regarding educational attainment, 33.9% had a university degree, 51.5% had completed secondary education (the majority of these participants had finished an apprenticeship), 5.1% had finished primary school, 3.4% had no degree, 3.4% had a degree not listed, and 1.7% did not answer this question.

In addition to the participants, all course instructors (*N* = 8, 3 females) completed the evaluation form.

### Participants’ and course instructors’ evaluations of the intervention

Overall, the course was rated positively by the participants (*M* = 3.9, *SD* = 1.1) and course instructors (*M* = 4.3, *SD* = 0.5). Regarding its comprehensibility, participants and course instructors evaluated the course very positively (*M =* 4.5, *SD* = 1.0 and *M* = 4.3, *SD* = 0.7, respectively). The ratings of the courses’ atmosphere were comparatively high (participants: *M* = 4.3, *SD* = 1.1; course instructors: *M* = 4.5, *SD* =0.5). Participants rated the opportunity to openly discuss illegal issues particularly high (*M* = 4.6, *SD* = 1.0), and the course instructors also provided high ratings on this measure (*M* = 4.3, *SD* = 0.7). As displayed in Table 3, compared to the ratings of the phase 1 participants, the ratings of the phase 2 participants tended to be higher across all general items. These differences were statistically significant for the overall evaluation and the evaluation of comprehensibility.

**Table 3:** General course evaluations from the participants of implementation phases 1 and 2

**How do you evaluate the course…**	**Phase 1 participants**	**Phase 2 participants**	**Mann–Whitney-*U *test**	
	**(*n*** **= 31)**	**(*n*** **= 28)**		
	***M (SD)***	***M (SD)***	***U***	***p***
…overall	3.6 (1.1)	4.1 (1.0)	306.0	.041
…regarding its comprehensibility	4.2 (1.2)	4.8 (0.5)	258.5	.018
…regarding the atmosphere	4.1 (1.2)	4.5 (0.9)	290.5	.100
…regarding the possibility to openly discuss illegal issues	4.4 (1.1)	4.8 (0.7)	302.5	.100

Items were answered on a scale ranging from 0 (not good at all) to 5 (very good).

Table 4 summarises the participants’ and course instructors’ evaluations of the different course components. Participants particularly appreciated the analysis of the consumption diary (*M* = 4.1, *SD* = 1.1), which was usually accompanied by an extensive group discussion involving exchanges of experiences between the participants. With the exception of “handling of social risk situations”, participants and course instructors provided high ratings of all the modules that aimed at developing concrete, personal strategies for handling problems that can occur during smoking cessation. Course instructors also considered the carbon monoxide measurement as helpful for the participants. Of all the course components, the Quit Teams which were an optional element received the lowest ratings from both the course instructors (*M* = 3.3, *SD* = 1.0) and the 38 participants who indicated that they have been a member of such a Quit Team (*M* = 2.4, *SD* = 1.7).

**Table 4:** Course participants’ and course instructors’ evaluations of the course components

**How helpful were the following components for you/for the participants?**	**Participants**	**Course instructors**
	**(*n*** **= 56)**	**(*n*** **= 8)**
	***M (SD)***	***M (SD)***
Information evening	3.6 (1.5)	4.3 (1.0)
Quiz	3.3 (1.4)	4.0 (1.3)
Pro & Con	3.5 (1.4)	4.0 (0.8)
Analysis of consumption diary/group discussion	4.1 (1.1)	4.3 (0.9)
Finding alternative rituals	3.8 (1.3)	4.4 (0.5)
Dealing with withdrawal symptoms	3.8 (1.4)	4.4 (0.5)
Goal setting	3.6 (1.4)	4.0 (0.8)
Handling of high-risk situations	3.8 (1.2)	4.3 (0.5)
Dealing with craving	3.7 (1.2)	4.6 (0.5)
Adaptation of personal strategies	3.4 (1.3)	4.0 (0.9)
Dealing with lapses	3.7 (1.3)	4.1 (0.6)
Handling of social risk situations (Saying “No”)	3.2 (1.4)	3.6 (0.9)
Preserving achievements	3.4 (1.3)	3.6 (0.9)
Participants’workbook	3.2 (1.4)	3.6 (1.0)
Consumption diary	3.3 (1.4)	3.4 (0.9)
Carbon mononxide measurement	3.7 (1.5)	4.5 (0.8)
Quit Team^1^	2.4 (1.7)	3.3 (1.0)

Items were answered on a scale ranging from 0 (not helpful at all) to 5 (very helpful).

^1^Only the ratings of the 38 participants who were member of a Quit Team are displayed.

## Discussion

This study describes the development and content of the first integrative group cessation program for co-smokers of tobacco and cannabis. The program was developed after a preliminary study revealed that both experts and co-smokers of cigarettes and cannabis demanded combined interventions to address simultaneous tobacco and cannabis cessation. This result is consistent with previous theoretical discussions [1,26,27].

During the preliminary study, experts stressed the multi-dimensional relationship between tobacco and cannabis use that is particularly evident during cessation attempts. Many consumers reported experiencing the balancing effect mentioned by the experts (i.e., the increased use of the other substance after quitting the first substance). However, among the queried co-smokers, there was only a modest level of readiness to quit tobacco and cannabis simultaneously and of readiness to participate in an ISCP that addressed both substances at the same time. Many co-smokers were not aware of the relationship between tobacco and cannabis use or the harmful physical health consequences of smoking cannabis. This lack of awareness might explain the modest level of readiness to quit simultaneously. To overcome this lack of awareness and knowledge, the interdisciplinary expert team developed a participant recruitment strategy that was an integral part of the ISCP.

The recruitment for the courses was a success because 77 participants were recruited, and seven courses were accomplished within a relatively short time frame of nine months. This success may be attributable to two factors. First, course participation was free of charge because the ISCP was still in development. In Switzerland, participants are usually required to pay for their own participation in tobacco cessation programs. Thus, participation rates may change when the program cannot be offered free of cost. Second, after the press release and a report that appeared in a common free commuter newspaper at the beginning of the first implementation phase, participation rates for the information evening and the course itself were especially high. Because this strategy cannot be applied regularly, it is necessary to switch to appropriate alternatives in the future. In Switzerland, these alternatives could include regular advertisements in the two most common commuter newspapers, which reach a large part of the population.

According to their evaluations of the course, both the participants and the course instructors found the ISCP highly acceptable. Because the items measuring the general acceptance of the course were rated more positively by the participants of the second implementation phase, a course length of six, rather than five, sessions may be more appropriate. The finding that participants especially appreciated the group discussions indicates that group settings are appropriate for targeting co-smokers of tobacco and cannabis. Furthermore, participants and course instructors valued those course modules that aimed at developing strategies which can be applied when quitting tobacco and cannabis use. Additionally, the course instructors considered the carbon monoxide measurements to be helpful. Thus, these measurements should remain part of the intervention when the scientific evaluation is completed and biochemical validation is not needed any longer.

The Quit Teams were the least appreciated course element and only two thirds of the participants engaged in a Quit Team. Possibly the participants felt no need of this optional buddy support system because the group setting provided sufficient social support. Thus, exclusion of the element of Quit Teams could be considered for a future version of the program. This conclusion is supported by two studies which show that buddy systems provide an additional benefit in an individual smoking cessation setting [43] but not in a group setting [44].

The ISCP developed in this study combines two substances in one cessation program and connects professionals from general health provider services and psychiatric services. Consequently, the professionals from these services will learn and potentially benefit from their complementary knowledge and experiences. However, beyond this intervention, policy makers should be sensitive to the issue of tobacco and cannabis co-use. Furthermore, the treatment of co-use should be implemented in the health care system and should be covered by existing basic health insurance. The public health approach of Screening, Brief Intervention, and Referral to Treatment (SBIRT) could be applied to the co-use of tobacco and cannabis. Thus, screening for tobacco use in primary care settings could be extended to include screening for co-smoking. Depending on the severity of co-smoking and the willingness to quit, practitioners could then provide information and advice and refer co-smokers to targeted interventions such as the one presented here. Proactive strategies like this may be capable of reaching a broad range of co-smokers and prevent the intake problems that the ISCP might face when it will be conducted without the media interest that surrounded its first implementation. Historical precedents, such as ignoring cannabis in tobacco cessation programs and vice-versa and the use of cigarettes as reinforcers in psychiatry [24,25], will hopefully become issues of the past.

However, the co-use of tobacco and cannabis should not only be addressed in treatment but also in prevention. According to the findings of our preliminary study, there is a lack of knowledge about the relationship between tobacco and cannabis among co-smokers. It is likely that smokers who only use tobacco or cannabis are not aware of this issue, and it is possible that increased awareness would help to prevent the initiation of the use of the second substance among these smokers. Thus, information about the problems associated with co-smoking should be spread, especially among adolescents and young adults.

One limitation of this study is that the online survey of former and active co-smokers was conducted using a convenience sample with a wide age range, those data are based on self-reports and retrospective cessation attempt reports. Furthermore, it is difficult to disentangle the reasons for the differences in the general evaluations of the course between the first and the second implementation phases. Course length may be one reason, but other factors, such as the number of participants in the courses and the identities of the course instructors, also varied between implementation phases.

Currently, we are conducting a thorough feasibility study on the ISCP and expect to have the results of the follow-up data in the autumn of 2013. More details on this feasibility study are provided in the study’s entry at Current Controlled Trials (ISRCTN15248397).

## Conclusions

The proposed intervention for co-smokers of tobacco and cannabis is important because it is the first group cessation program targeting these two interrelated substances simultaneously. The developed ISCP integrates the opinions of both users and experts, established therapeutic principles, and the strategies of former tobacco and cannabis cessation programs. This intervention also takes into account reasonable concepts and ideas that have emerged from on-going discussions about the underlying mechanisms and relationships between cannabis and tobacco use, such as the common route of administration [1]. To prevent one substance from acting as a behavioural cue for the other [32], participants are expected to quit both substances simultaneously. Preliminary results show that the developed ISCP was well accepted among the participants and the course instructors. The group discussions and the development of personal strategies for the dual cessation of tobacco and cannabis use were particularly appreciated. These promising results also underline the high acceptance of the ISCP among the co-smokers, who, for the first time, had access to a group intervention especially targeted to them.

In the near future, the presented ISCP will be evaluated for feasibility and initial efficacy.

## Competing interests

The authors declare that they have no competing interests.

## Authors’ contributions

JB analysed the quantitative data from the preliminary study, contributed to program development, and wrote the first draft of the manuscript. IH performed the data collection for the preliminary study, analysed and interpreted the qualitative data, and revised the manuscript. OB, MS, AH, and AK contributed to program development and revised the manuscript. MPS designed the study, supervised the data collection and analyses, contributed to program development, and revised the manuscript. All authors have approved the final version of the manuscript.
